# Genome and plasmid diversity of Extended-Spectrum β-Lactamase-producing *Escherichia coli* ST131 – tracking phylogenetic trajectories with Bayesian inference

**DOI:** 10.1038/s41598-019-46580-3

**Published:** 2019-07-16

**Authors:** Sofia Ny, Linus Sandegren, Marco Salemi, Christian G. Giske

**Affiliations:** 1 Division of Clinical Microbiology, Department of Laboratory Medicine, Karolinska Institutet, Alfred Nobels allé 10, 141 52 Huddinge, Stockholm Sweden; 2 Public Health Agency of Sweden, Nobels väg 18, 17182 Solna, Stockholm Sweden; 3 Department of Medical Biochemistry and Microbiology, Uppsala University, BMC, Box 582, Husargatan 3, 75123 Uppsala, Sweden; 4 Department of Pathology, University of Florida. Emerging Pathogens Institute, University of Florida, P.O. Box 100009, Gainesville, Florida 32610-0009 USA

**Keywords:** Phylogenetics, Bacterial genetics, Bacterial infection

## Abstract

Clonal lineages of ESBL (Extended-Spectrum β-Lactamase)-producing *E*. *coli* belonging to sequence type 131 (ST131) have disseminated globally during the last 30 years, leading to an increased prevalence of resistance to fluoroquinolones and extended-spectrum cephalosporins in clinical isolates of *E*. *coli*. We aimed to study if Swedish ESBL-producing ST131 isolates originated from single or multiple introductions to the population by assessing the amount of genetic variation, on chromosomal and plasmid level, between Swedish and international *E*. *coli* ST131. Bayesian inference of Swedish *E*. *coli* ST131 isolates (n = 29), sequenced using PacBio RSII, together with an international ST131 dataset showed that the Swedish isolates were part of the international ST131 A, C1 and C2 clades. Highly conserved plasmids were identified in three clusters although they were separated by several years, which indicates a strong co-evolution between some ST131 lineages and specific plasmids. In conclusion, the tight clonal relationship observed within the ST131 clades, together with highly conserved plasmids, challenges investigation of strain transmission events. A combination of few SNPs on a genome-wide scale and an epidemiological temporospatial link, are needed to track the spread of the ST131 subclones.

## Introduction

Few antibiotic resistant clones have generated as much interest as *Escherichia coli* ST131. In recent years several papers have described its emergence, evolution, and molecular epidemiology globally^1–9^. The interest is justified since ST131 and especially its successful subclone C2/H30-Rx (hereafter referred to as C2) is the dominating multidrug-resistant (MDR) *E*. *coli* lineage in many parts of the world and is overrepresented among human infections. Its rapid dissemination has been suggested to be partially due to the advantageous combination of fluoroquinolone resistance as well as IncF plasmids encoding the extended-spectrum β-lactamase (ESBL) -gene *bla*_CTX-M-15_^2,10–12^. The frequent association with IncF-plasmids encoding *bla*_CTX-M-15_ adjacent to an active ISEcp1 transposase suggests that this clone also serves as a main driver of the spread of the *bla*_CTX-M-15_ gene to other strains and species^12^.

The emergence of the successful ST131 C2 clade, carrying fluoroquinolone resistance and the *bla*_CTX-M-15_ gene, started in the mid-1980s and was followed by a rapid international clonal expansion during the 1990s^1–3,7^. However, it was not until 2008, when MLST was broadly introduced, that the clone was simultaneously described in several places^4,5,13^. Since then the emergence of the ST131 subclone C1/H30-R (referred to as C1) with *bla*_CTX-M-27_ has also been described^14,15^. Most genetic variation within the ST131 group is caused by recombinational events including traits such as virulence, antibiotic resistance and other accessory traits^1,3,8^. Studies on the arrangement of resistance genes in ST131 isolates could help to better understand the spread, selection due to resistance and the evolution of the accessory genome in this internationally circulating clone.

No ST131 isolates from Sweden have thus far been part of global phylogenetic studies. Sweden offers a different perspective, with low antibiotic pressure and low prevalence of resistant bacteria in animals and humans, compared to other countries represented in previous studies^16^. International travel to high prevalence regions is a known risk factor for being a community carrier of ESBL-producing *E*. *coli* in Sweden^17–19^. The ST131 subclone C2 was estimated to a have a prevalence of 0.3% in Swedish community carriers in a nationwide study^19^.

We sought to elucidate if the Swedish ST131 population is mostly shaped by constant new influx of international isolates or if it is dominated by national spread from a single introduction. Given the low prevalence of ESBL-producing and low antibiotic selection pressure in Sweden, we hypothesised that ST131 isolates from Swedish carriers and patients would cluster with international clones in several different clades, if observed cases are mainly due to constant influx. This hypothesis was investigated by constructing a phylogeny of ESBL-producing ST131 isolates from i) patients with bloodstream infections and ii) non-symptomatic community carriers in Sweden in relation to previously published international ST131 isolates^3^. Plasmid similarities within clusters were used to study if there were specific plasmids associated with closely related isolates, which would provide an additional level of resolution during outbreaks. Swedish ST131 isolates (n = 29) were sequenced using long-read single molecule real-time sequencing (PacBio), allowing assembly of both chromosomes and plasmids. Bayesian phylogenetic inference was used to investigate the evolutionary patterns of Swedish ST131 isolates and their relationship to previously published genomes (n = 91) from strains circulating internationally^3^.

## Results

### Dataset generation and assembly

The selected ESBL-producing *E*. *coli* ST131 isolates were from patients with bloodstream infection (n = 20) and from non-symptomatic community carriers (n = 9) in Sweden (Supplementary Table S1, deposited in NCBI under BioSample accession SAMN10839615 to 43). The isolates were selected based on heterogeneity in their phenotypic resistance pattern, *bla*_CTX-M_ gene type and plasmid replicon type to achieve a broad representation of the ST131 population in Sweden.

The published ST131 dataset consisted of a mix between non-ESBL and ESBL-producing isolates from 6 countries on 4 continents^3^. SNP calling, on 120 isolates, was made on the 79% shared dynamic core genome with the reference strain (EC958), and the output alignment contained 13,351 SNPs. After removing recombinant regions 3,297 non-recombinant SNPs remained for Bayesian phylogenetic analysis

### Bayesian molecular clock calibration and mutation rate

The correlation coefficient for the root-to-tip genetic divergence versus time in the TempEst analysis (R^2^ = 0.25) indicated a weak linear relationship between accumulated mutations and sampling time, but suggested that enough signal may be present to calibrate a relaxed molecular clock. Bayesian model comparison through Bayes factors confirmed the uncorrelated relaxed clock model and with Bayesian Skyline tree prior was the best fitting for the alignment (Supplementary Table S2). The median molecular clock rate was estimated to 8.5 * 10^−4^ (95% HPD interval 6.6 * 10^−4^ to 1.03 * 10^−3^) mutations per site per year which translates to approximately 2.7 mutations per year per genome (Supplementary Table S2), with a median coefficient of variation for the clock rate of 0.41 (95% HPD interval 0.30 to 0.54).

### Several introductions have shaped the swedish ST131 population

**Figure 1:**
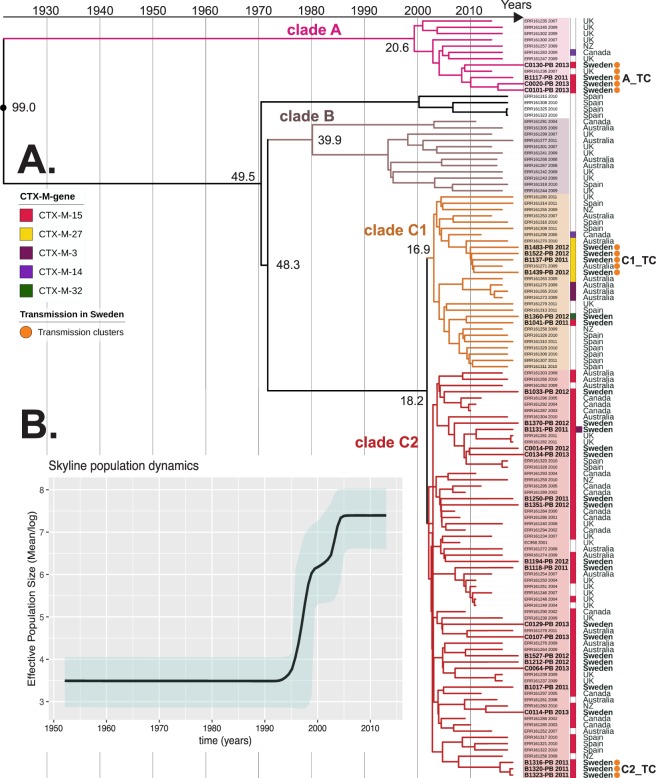
(**A**) BEAST ST131 phylogenetic tree with Swedish and international isolates, (n = 121) constructed from an alignment of 3,297 non-recombinant SNPs. (**A**) The ST131 tree consists of five clades A, B, C1 and C2 including an additional clade separation before the (**A**,**C**) clades. Visualized metadata includes *bla*_CTX-M_ resistance gene (marked with coloured squares) and country of isolation. Three suspected transmission clusters clade A_TC, clade C1_TC and clade C2_TC are marked with orange circles. Swedish isolates starting with B were isolated from patients with bloodstream infections while isolates starting with (**C**) were from community carriers. (**B**) Bayesian skyline plot of estimated changes in the effective population size (N*e*) over time with 95% CI displayed in blue.

The combined phylogeny of the international and Swedish ST131 showed a scattered distribution of the Swedish isolates in clades A, C1 and C2 (Fig. 1). No isolates from Sweden belonged to clade B. A fifth clade, with isolates from Spain, which separated from the B/C clade in the beginning of the 1960s was also present in the tree branch, albeit with low statistical support (0.4 posterior probability). All other major clade separations had a branch support of 1.0. The major clade separations between A and B was suggested to have occurred in 1914 (95% HPD 1866 to 1952), between B and C 1964 (95% HPD 1950 to 1979) and between C1 and C2 in 1994 (95% HPD 1991 to 1997) (Fig. 1). The skyline plot showed a sharp increase in the estimated effective population size during the 1990s which levelled out after 2005 (Fig. 1).

The distribution of the Swedish isolates showed three suspected national transmission clusters, where Swedish isolates clustered together under a Most Recent Common Ancestor (MRCA), located in the clades A (five isolates), C1 (five isolates) and C2 (three isolates), marked with circles in Fig. 1.

**Figure 2:**
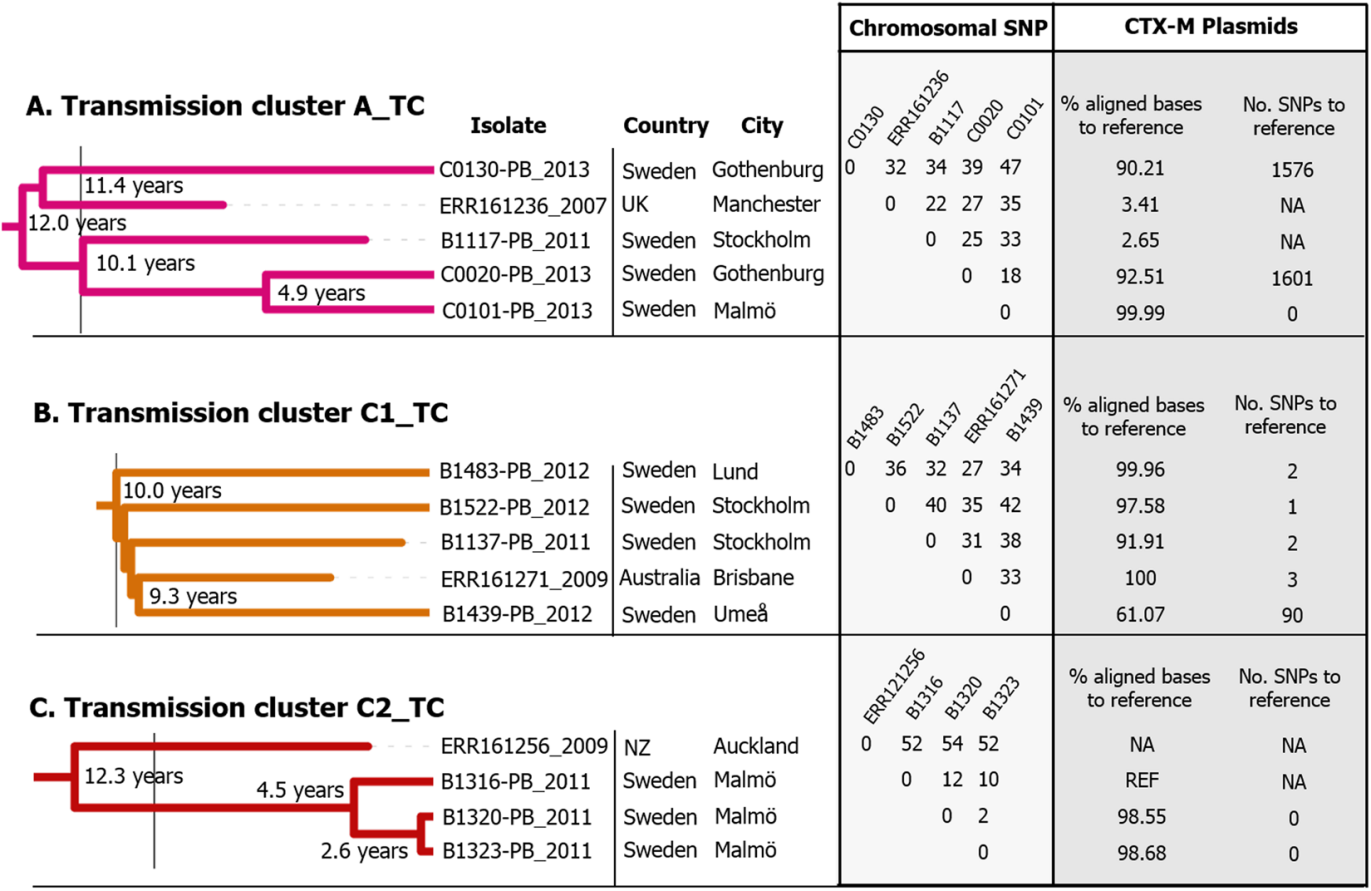
Chromosomal and plasmid differences in identified Swedish transmission clusters, in clade A (A_TC), C1 (C1_TC) and C2 (C2_TC). Data included for each strain are; place of isolation (country and city), pairwise SNP differences on chromosomal level (chromosomal SNP), percentage aligned bases between *bla*_CTX-M_ plasmids and a reference plasmid (% aligned bases to reference) and *bla*_CTX-M_ plasmid SNP differences to reference plasmid (No. SNPs to reference). Reference plasmid for clade A_TC GenBank: HG530657.1 (size:111,741 bp) and clade C1_TC GenBank: CP021871.1 (size 134,899 bp). For C2_TC an internal reference plasmid (B1316-PB_2011, size: 101,922 bp) was used. NA = non applicable, UK = United Kingdom, NZ = New Zealand.

The number of pairwise chromosomal SNPs separating the transmission cluster in clade A (A_TC) were 18 to 47, in clade C1 (C1_TC) 27 to 42 and in clade C2 (C2_TC) 2 to 12 (Fig. 2, Supplementary Table S3). The median time estimations to MRCA was 12.0 years for the A_TC, 10.0 years for the C1_TC and 4.5 years for the C2_TC. The five isolates in clade A_TC were isolated at three different locations in Sweden; Stockholm, Malmö and Gothenburg and one was isolated in the UK. The five isolates in the C1_TC, carrying *bla*_CTX-M-27_, were isolated at three different places in Sweden that are distantly separated geographically (Umeå, Stockholm and Lund) and one isolate was part of the international dataset and isolated in Australia. All isolates in the C2_TC were isolated in the Swedish city Malmö (Fig. 2).

### Conserved plasmids identified in all suspected transmission clusters

**Figure 3:**
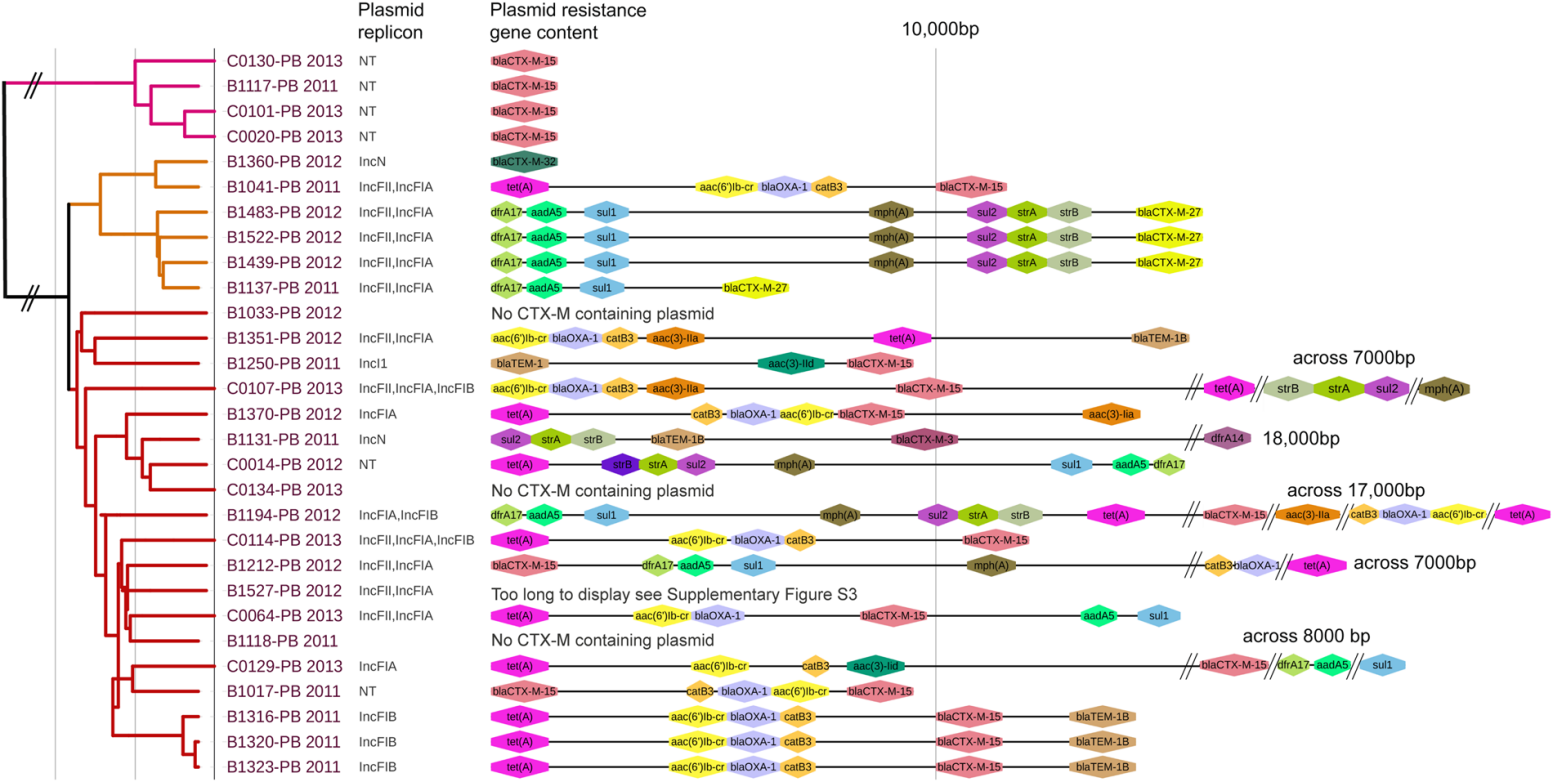
Comparison of resistance gene placement on *bla*_CTX-M_ encoding plasmids among Swedish ST131 isolates (n = 29). For five of the isolates the sequences were cut to accommodate all resistance genes, the actual length of the region harbouring these genes is written next to the sequence. Isolates starting with B were from patients with bloodstream infections while isolates starting with C were from community carriers. NT = Non-Typable.

The three suspected Swedish transmission clusters, in clade A, clade C1 and clade C2, had similar arrangements of their resistance genes and a common MRCA close in time (<12 years) within each cluster (Figs 2 and 3, Supplementary Tables S5–S7).

In clade A_TC all four Swedish isolates included a plasmid with a single *bla*_CTX-M-15_ gene (all around 127 kb) while the additional resistance genes were located on a second plasmid (Fig. 3, Fig. S1 Supplementary Tables S4 and S5). Alignment and SNP calling from shared regions displayed above 90% similarity between three of the clade A_TC plasmids and a plasmid isolated in 2006 or 2007 in Germany (Fig. 2, reference plasmid HG530657.1 not included in the figure)^20^. One isolate from a Swedish community carrier (C0101-PB_2013) only lacked 4 nucleotides compared to the reference plasmid and had 0 SNPs in shared regions. Two Swedish plasmids in the A_TC cluster differed from the reference with around 1600 SNPs respectively which were distributed over the entire plasmid (Fig. 2, Supplementary Table S5 and Fig. S2). Isolate B1117-PB_2011 and the UK isolate in clade A_TC contained completely different plasmids and the B1117-PB_2011 only shared the region containing the *bla*_CTX-M-15_ gene with the other plasmids in the cluster. Regarding the plasmid in isolate B1117-PB_2011, NCBI BLAST identified two very similar plasmids, CP031903.1 and LT985295.1, both displaing 99% query coverage with 99.94% identities.

The four Swedish isolates in clade C1_TC had plasmids with the same resistance gene arrangement, except for one strain (B1137-PB_2011) that had lost part of the plasmid containing the resistance genes *mph*(*A*), *sul2*, *strA* and *strB* (Fig. 3). Alignment and SNP calling from shared regions for the clade C1_TC plasmids (97 to 150 kb in size) to a reference plasmid isolated in Germany in 2010 showed between 61 and 99% aligned bases (Fig. 2 (reference plasmid CP021871.1 not included in the figure), Supplementary Table S6)^21^. The plasmids differed from each other (including the reference plasmid) with 1 to 90 SNPs. The most diverse plasmid (B1439-PB_2012) differed with approximately 90 SNPs that were all situated in a 3594 bp recombination event (Fig. 2, Supplementary Table S6 and Fig. S2). The B1439-PB_2012 plasmid was smaller compared to the reference plasmid (87,000 bp vs 144,000 bp) and uncalled regions in the alignment corresponded to genes in the reference plasmid encoding different transposases, conjugative transfer systems and helicases.

The transmission clade C2_TC had the same arrangement of resistance genes on their plasmids (98 to 102 kb) (Fig. 3). No closely related plasmid was identified in the NCBI search (all less than 50% query coverage) so B1316-PB_2011 was used as reference. All plasmids had above 98% aligned bases and the SNP calling showed no variation in shared regions. Seven deletions were identified accounting for 244 to 1476 bp in total (Supplementary Table S7).

### Heterogeneous arrangement of resistance genes between plasmids and chromosome

The distribution of *bla*_CTX-M_ genes in the Swedish dataset showed that *bla*_CTX-M-15_ was common in clade C2 but also appeared in the A and C1 clades while the *bla*_CTX-M-27_ exclusively appeared in C1 (Fig. 1). The *fimH* types for the Swedish isolates were *fimH41* in clade A and *fimH30* in the rest of the isolates. The organisation of resistance genes was overall heterogeneous in their distribution between plasmids and chromosomal regions both within and between isolates. The majority of the resistance genes were located in the *bla*_CTX-M_ encoding plasmid, except for two isolates in clade A (B1117-PB_2011 and C0101-PB_2013) (Fig. 3 and Supplementary Fig. S1). Eight isolates had more than one resistance plasmid (Fig. S1). The number of unique resistance genes were between 1 and 14 per isolate. One plasmid (B1527-PB_2012) included five copies of the same resistance cassette (confirmed with a combination of long-read PacBio sequencing and coverage analysis using IonTorrent short-read data) Supplementary Fig. S3.

In total eight isolates had resistance genes located in the chromosome and two isolates had only chromosomally encoded resistance genes (C1118-PB_2011, C0134-PB_2013) (Fig. S4). One isolate (B1017-PB_2011) had its resistance genes distributed over two plasmids and the chromosome. All chromosomally located ESBL-resistance was due to *bla*_CTX-M-15_ insertions and in four cases it was the only resistance gene found on the chromosome (Fig. S4).

## Discussion

We describe the relationship between the Swedish ESBL-producing *E*. *coli* ST131 population and the international ST131 lineage. The results demonstrate that both national transmission clusters and introductions of international clones contribute to the ST131 population structure in Sweden. We also show close relationship between international clonal clusters, chromosomes and plasmids, in the ST131 A and C1 clades, without them being part of a national transmission chain. This finding illustrates the challenges when investigating outbreaks with conserved clonal lineages, as few methods can provide appropriate resolution to draw conclusions on strain transmission based on molecular typing data.

### Evolutionary history and clade separation in line with previous publications

The presented ST131 tree (Fig. 1) including Swedish and international isolates showed the same topological distribution of clades as previously published phylogenetic trees^1–3,7,9^. Our results are also in line with a previous BEAST analysis favouring the same substitution model, clock model and tree prior^1^. Since we used an external dataset that was also included in the study by Zakour *et al*., it is not surprising that our results are similar^1,3^. We saw slightly different values for time point clade separation (C1/C2 clade 1994 in our study versus 1986 for Zakour *et al*.) and mutation rate (SNP/site/year of 8.4 * 10^−4^ in our study vs 4.39 * 10^−7^ for Zakour *et al*.). These values are naturally connected since a higher mutation rate (our study) leads to more variations in a shorter time period and therefore a later divergence date. The large difference in mutation rate between the studies could be due to different methods in removing recombination, however very similar SNP alignment length after removing recombination suggests otherwise (3,297 bp in our study vs 3,779 bp for Zakour *et al*.). The mutation rate estimation, in our study, translates to approximately 2.7 fixated SNPs/year/genome which means that around 5 SNPs can be expected to differ between two isolates sharing a MRCA one year back in time. The number (2.7 SNPs) could be perceived as high, but importantly this is the number of SNPs in the entire genome (5 Mb), excluding recombinant SNPs and therefore an estimate to be used with caution. The estimated changes in effective population size (Skyline plot Fig. 1) show similar variations over time as previously suggested^1^. Considering that the first report of *bla*_CTX-M-15_ was published in 2001 it is fascinating to see that the population increase was already ongoing at that time^22^. It also likely illustrates the powerful driving force of fluoroquinolones like ciprofloxacin that was introduced on the market 1987 and has been described by many as one of the most important factors for the success of the ST131 C2 lineage^1,2,23^. The skyline model suggests that a plateau was reached in 2005, after which the ST131 population entered a steady state where the population did neither decline nor increase (Fig. 1). This finding is in agreement with Swedish surveillance data, where the proportion of ST131 in ESBL-producing *E*. *coli* urinary tract infection has been stable since surveillance started in 2007^24^.

The observations herein suggest that a constant influx of ST131 has taken place to the Swedish ST131 population, since the Swedish isolates were mixed with the international sublineages in the phylogeny (Fig. 1). We also observed local national transmission clusters. One important aspect is that our dataset was not designed to quantify the burden of international import versus national spread of ESBL-producing ST131 in Sweden. To do this a larger and differently selected dataset would be needed. National transmission could therefore be the largest contributor to infections with ESBL-producing *E*. *coli* ST131 in Sweden.

### High stability of plasmids within the major observed transmission clusters

Because of the clonal nature of the isolates in the different ST131 clades it can be challenging to determine which isolates that have spread locally versus international influx. This was seen in both the A and C1 clade where strains clustering together was isolated at different places in Sweden and the world (UK and Australia) and therefore represent a tight clonal lineage rather than a direct transmission chain. Including more data from the international ST131 population could potentially have increased resolution within transmission cluster A and C1 which would be interesting clusters to examine in future analyses.

In clade A the cluster containing five isolates had limited chromosomal variation and three of the isolates had very similar plasmids (Fig. 2). These three Swedish plasmids showed high resemblance to a plasmid isolated from a patient in Germany in 2006 or 2007 (HG530657.1) that we used as reference for the plasmid SNP call (over 90% of sequence shared)^20^. One plasmid turned out to be almost identical to the German reference sharing 99.99% of its sequence and containing 0 SNPs (Clade A Fig. 2). Considering that the Swedish isolate was from a community carrier and isolated 5–6 years after the German isolate, it shows remarkable stability. The other two plasmids in the cluster had accumulated SNPs over time (around 1,600 SNPs each) but still shared over 90% of the sequence, and the variations seen were likely not due to recombination since an even SNP accumulation was seen across the genome (Supplementary Fig. S2). The Swedish B1117-PB_2011 isolate had completely different plasmids that only shared the region containing the *bla*_CTX-M-15_ gene itself with the other plasmids (Fig. 2). This gives insight into how unpredictable the plasmid composition of closely related strains can be. One likely explanation is that the highly mobile cassette harbouring the ISEcpI and *bla*_CTX-M-15_ genes has moved between plasmids. This is also a likely explanation to why we saw high presence of *bla*_CTX-M-15_ with chromosomal location in our Swedish dataset. Since one of the isolates in the cluster (Fig. 2) was isolated in the UK, and the Swedish isolates were from very different geographical regions, this clade A clonal lineage is likely circulating internationally among community carriers but also causes clinical infections.

The BEAST analysis showed that the C1_TC had a MRCA around 10 years back in time (Figs 1 and 2). Since all isolates in this cluster, including one isolate from Australia, carried the same conserved plasmid, with only 1–3 SNPs or one recombination event, it must have been present already in the MRCA 10 years ago (Fig. 2, Supplementary Table S6)^14,15,21^. In addition, the Australian isolate just outside the cluster (ERR161270_2010 Fig. 1) also carried the same conserved plasmid (Supplementary Table S6). The conserved plasmid together with the limited chromosomal SNP differences (around 30–40 SNPs) in the C1_TC cluster indicate a strong co-evolution between the plasmid lineage carrying *bla*_CTX-M-27_ and the specific C1 ST131 clade as has been described previously^14,15^. A dataset including more representatives of the C1 *bla*_CTX-M-27_ clone might have given a better resolution and helped to separate the Swedish isolates into several clades.

The three Swedish C2_TC isolates only differed by 2, 10 and 12 SNPs on a chromosomal level and no SNPs were seen in the 98% shared plasmid sequence. All three isolates came from the same Swedish city and likely represents local spread. The C2_TC was the only transmission cluster identified as likely to be local for Sweden in this dataset.

## Conclusions

The evidence presented herein offers a deepened insight into the Swedish epidemiology of ESBL-producing ST131 and its close relationship to internationally disseminating ST131 clones. It is evident that the Swedish ST131 population is part of the international lineages and that several introductions combined with national transmission have formed the strain population. The clonal nature of the ST131 lineage, with highly conserved plasmids in some sub-lineages, complicates estimation of local circulation and transmission, and highlights the importance of temporospatial epidemiological links even in the genomic era. However, very close genetic relationships (a few chromosomal SNPs) could indicate a direct transmission even if the epidemiological link is unknown. Such small differences can only be detected by whole genome sequencing and not with traditional typing methods. Online tools and databases to analyse WGS data adapted for clinical microbiologists and public health workers could provide the possibility to detect outbreaks with clonal linages like ST131, two examples are BacWGSTdb and Enterobase^25,26^. The presence of plasmids, which were highly conserved over many years, in a widely disseminated clonal lineage illustrates that identical plasmids sequences are not a certain evidence of plasmid transmission. If a suspected plasmid transmission takes place in a hospital and identical plasmids are identified it still might not be a direct spread if the plasmid originates from one of these conserved lineages. Therefore, despite the emergence of sequencing technology that allows for rapid plasmid sequencing in the clinical settting, more work is still to understand the role of conserved plasmids in certain conserved clonal lineages, and how this phenomenon can impact inference about plasmid transmission events.

## Material and Methods

### Swedish clinical isolates

A total of 29 strains of *E*. *coli* ST131 isolates from patients with bloodstream infections (n = 20) and from non-symptomatic community carriers (n = 9) were selected from a larger dataset of ST131 (n = 177) from bloodstream infections and community carriers. The data collection and molecular typing was previously described^19^.

The isolates were selected from a Swedish nationwide collection of *E*. *coli* ST131 bloodstream isolates (n = 177) producing ESBL (2011–2012) and from a point prevalence study on community carriage (n = 16) of ESBL-producing *E*. *coli* (2013)^19^. We selected ST131 isolates from the community carriers and a subset of bloodstream isolates based on C2 status, phenotypic resistance profiles, *bla*_CTX-M_ types, and plasmid replicon type^2,19^. Based on these criteria we included isolates to represent the diversity, as well as representative isolates from two possible transmission clusters. The selected isolates had the ESBL genes *bla*_CTX-M-15_ (n = 23), *bla*_CTX-M-27_ (n = 4), *bla*_CTX-M-3_ (n = 1), *bla*_CTX-M-32_ (n = 1). The majority of isolates had different IncF plasmids (n = 25) (Supplementary Table S1).

### Sequencing and external international dataset

Genomic DNA was extracted using Qiagen genomic tip 500/G kit and sequenced with PacBio RSII (Pacific Biosciences) using one SMRT^M^ cell per isolate. For detailed data on the dataset see Supplementary Table S4. Hierarchical Genome Assembly Process (HGAP) was used to generate draft assemblies/genomes. Plasmids identified in transmission clusters in clade A, C1 and C2 were further closed using Flye assembler version 2.4.2 https://github.com/fenderglass/Flye^27,28^. Assembled genomes were deposited at NCBI under BioProject PRJNA517648 with BioSample accessions SAMN10839615 to 43. Isolate B1527-PB_2012 was also sequenced using Ion Torrent S5 XL (Thermo Fischer Scientific). A publicly available WGS Illumina dataset (FASTQ-files) of 91 *E*. *coli* ST131 was used as comparison and reference for the different subclones within ST131^3^. *FimH*, plasmid replicon and resistance genes were identified via the CGE website using FimType, PlasmidFinder and ResFinder respectively (https://cge.cbs.dtu.dk/services/) accessed on 2019-03-05^29^. All annotations were made in CLC Genomic Workbench v8.0.1 (Qiagen Bioinformatics, Redwood City, USA).

### SNP-call, alignments and recombination removal

Variation calling, mapping and, *de novo* assembly of the 121 genomes was done using CLC Assembly Cell Version: 4.4.2.133896 (Qiagen Bioinformatics). Isolate EC958 was used as reference for the chromosome SNP call because of it being a well sequenced (long and short read), assembled and closed genome from the C2 clade^30^. For the external international dataset the deposited FASTQ files were used while for the Swedish PacBio dataset the HGAP assembled genomes in the SNP-call were used. Minimum coverage, for FASTQ, was set to 10 and the SNP ratio cut-off for SNP support was set to 90%.

Alignment and SNP call of suspected transmission cluster plasmids was done with Snippy v4.0 (https://github.com/tseemann/snippy accessed on 2019-03-05). SNP distance matrixes were calculated using snp-dist v0.6 (https://github.com/tseemann/snp-dists accessed on 2019-03-05). Two reference plasmids were used for SNP-calling in transmission cluster A (HG530657.1, query coverage: 98%, identity 99.98%) and C1 (CP021871.1 query coverage: 100%, identity 99.99%) and identified by using NCBI nucleotide BLAST using the assembled plasmids as queries with the cut-offs >95% query coverage and >99% identity. For the transmission cluster in C2 an internal reference was used (B1316-PB_2011) since the closest match at NCBI (CP036244.1) had only 67% query coverage. Re-assembly with IonTorrent data of the resistance plasmid from isolate B1527-PB_2012 against the plasmid assembled from the PacBio data, was done using CLC Genomic Workbench v8.0.1 (Qiagen Bioinformatics). Gubbins version 2.3.1, with standard settings, was used to remove SNPs from recombinant regions^31^.

### Bayesian inference

The presence of enough phylogenetic signal in the ST131 aligned sequences was investigated by likelihood mapping analysis using IQ-TREE (http://www.iqtree.org/ accessed on 2019-03-05) and allowing the software to search for all possible quartets using the best-fitting nucleotide substitution model^32^.Temporal signal was assessed by plotting root-to-tip divergence versus sampling time with TempEst v1.5 (http://tree.bio.ed.ac.uk/software/tempest/) of the maximum likelihood (ML) tree including all aligned sequences^33^. ML tree was inferred with RaxML, using the best-fitting nucleotide substitution model bootstrapping (1,000 replicates) to assess statistical robustness for internal branching order in the phylogeny^34^. Bayesian phylogenetic inference was carried out with BEAST v1.10^35^. Path- and stepping stone sampling was used for the marginal likelihood estimation^36,37^. A Markov Chain Monte Carlo (MCMC) was run for 200 million generations, sampling every 20 million generations. Analysis files (xml) were generated in BEAUti using; collection year tip dating, site heterogeneity gamma model with 4 categories and ascertainment bias correction. Both strict and uncorrelated relaxed clock was tested with the following tree priors: constant size, exponential growth, GMRF Bayesian Skyride and Bayesian skyline with 3 groups. The substitution models HKY and GTR were also tested. Proper sampling of the Markov chain (Xml files are available upon request) was evaluated by calculating the effective sampling size (ESS) with Tracer 1.7; ESS values >200 for parameter estimates were considered acceptable^38^. Bayes factor (BF) was used in the estimation of best model fit for the dataset. TreeAnnotator was used to create Maximum Clade Credibility (MCC) trees with 10% burn in. Visualisation of trees and metadata was done using iTOL (https://itol.embl.de/ accessed on 2019-03-05)^39^. Bayesian skyline plot was generated in Tracer v1.6 and visualized in R v3.5.1. The posterior distribution of trees was summarized into the maximum clade credibility (MCC) tree with TreeAnnotator v1.8.4 (BEAST package) after 10% burn-in and the final tree edited with FigTree (http://tree.bio.ed.ac.uk/software/figtree/ accessed on 2019-03-05). All computations were performed on resources provided by the Swedish National Infrastructure for Computing (SNIC) at Uppsala Multidisciplinary Center for Advanced Computational Science (UPPMAX).

### Ethical approval and informed consent

This study was conducted in accordance with the Declaration of Helsinki and national and institutional standards and was approved by the research ethics committee “Regionala Etikprövningsnämnden i Stockholm, EPN” in Stockholm, Sweden (Record: 2012/1204-31/4). Written informed consent was obtained from all participants contributing with samples.

## Supplementary information

Supplementary information

Supplementary TablesS1-S7

## Data Availability

Additional data and analysis files produced during this study are available upon request. Sequence data were deposited at NCBI under BioSample accession SAMN10839615 to 43.
