# Supplementary material for: Genome and plasmid diversity of Extended-Spectrum β-Lactamase-producing Escherichia coli ST131 – tracking phylogenetic trajectories with Bayesian inference
Source: Sci Rep. 2019 Jul 16;9:10291. doi: 10.1038/s41598-019-46580-3 (PMC6635401; doi:10.1038/s41598-019-46580-3)
Supplement: Supplementary file 1 — Supplementary information [file 41598_2019_46580_MOESM1_ESM.pdf]

Supplementary information

Genome and plasmid diversity of Extended-Spectrum β-Lactamase-producing *Escherichia coli* ST131 – tracking phylogenetic trajectories with Bayesian inference

Authors
Sofia Ny, Linus Sandegren, Marco Salemi, Christian G. Giske

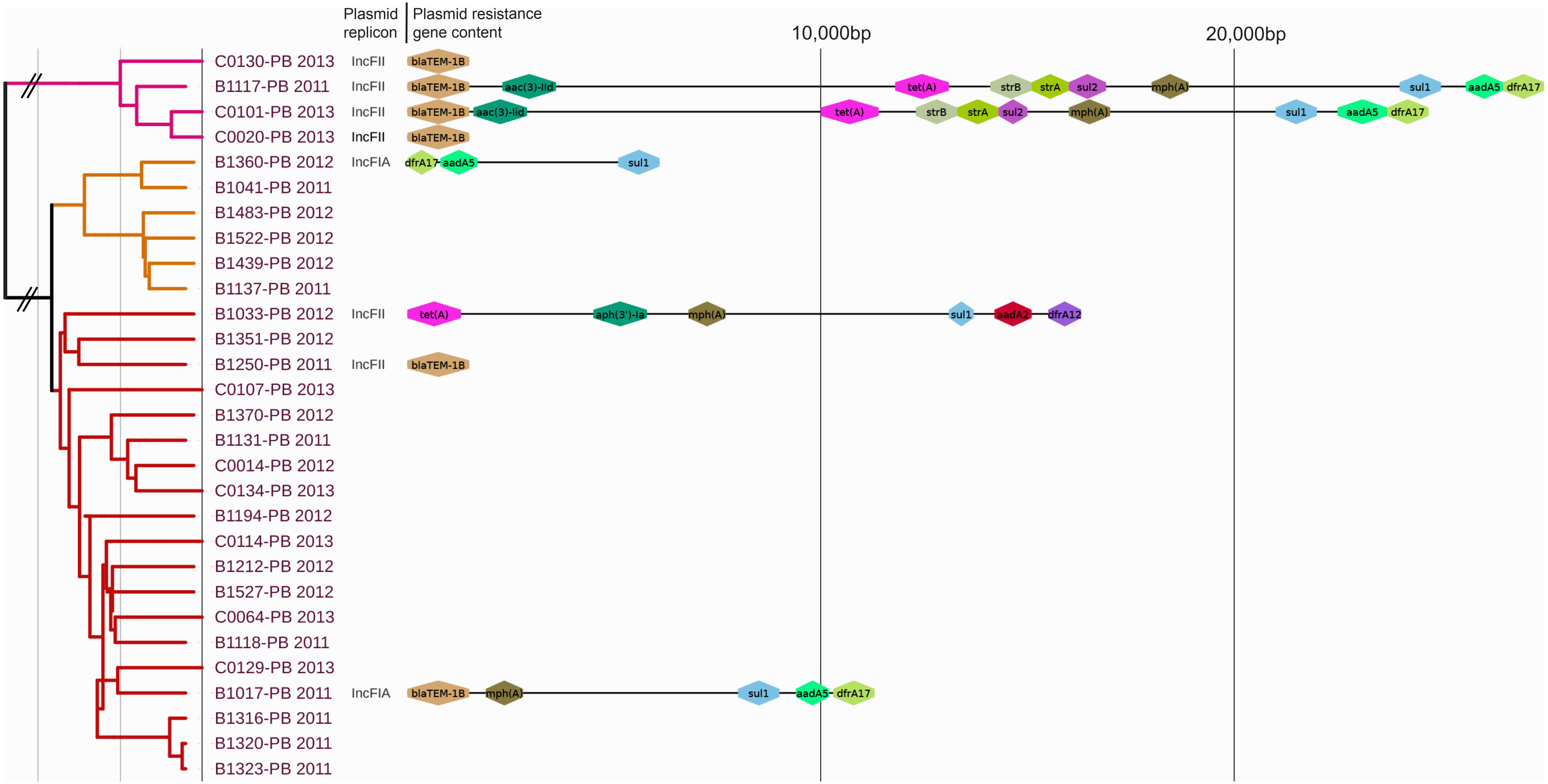

Figure S1. Comparison of resistance gene location on non- *bla*$_{\text{CTX-M}}$ plasmids in Swedish ST131 isolates. In total eight isolates had a second plasmid containing resistance genes apart from the *bla*$_{\text{CTX-M}}$ encoding plasmid.

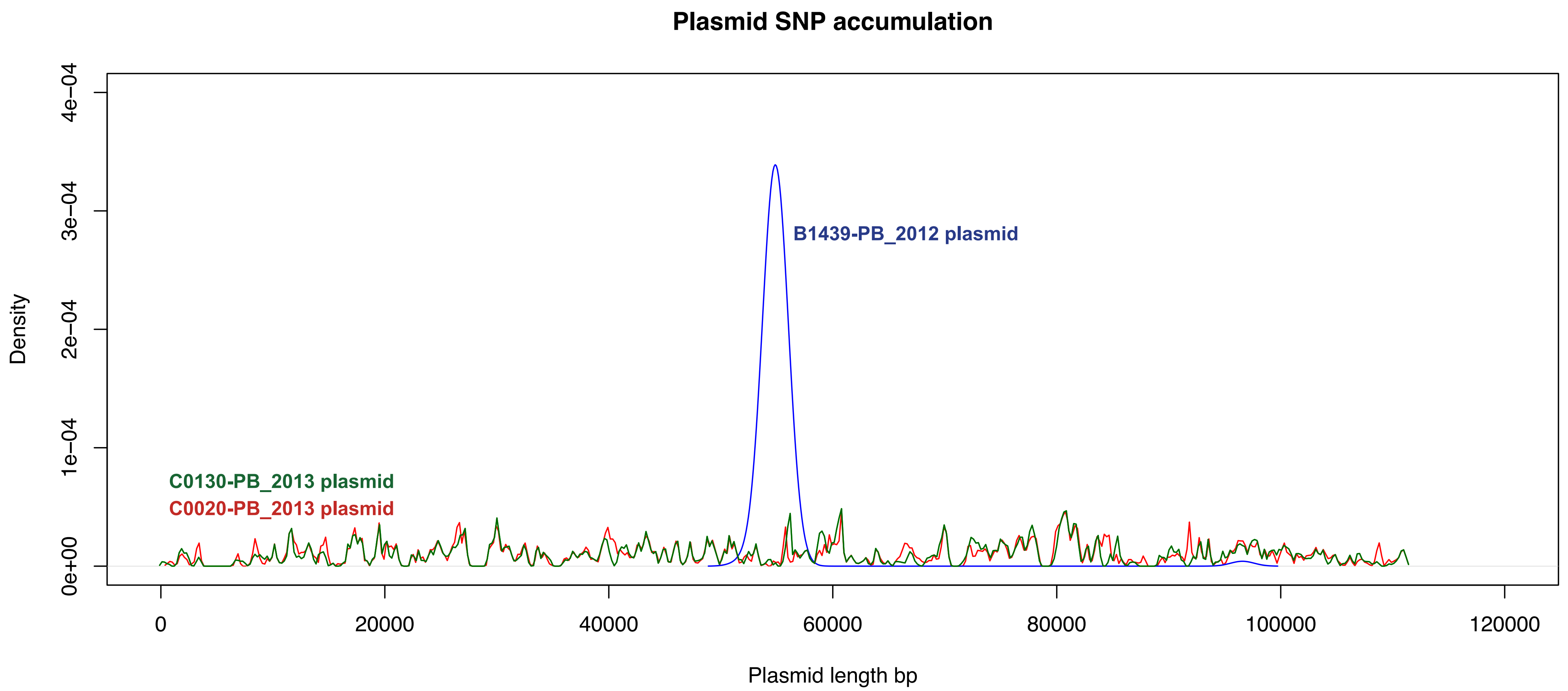

Figure S2. SNP distribution over *bla*$_{CTX-M}$ plasmids for isolates C0020-PB_2013 (126,263 bp), C0130-PB_2013 (127,016 bp) and B1439-PB_2012 (87,020 bp). In B1439-PB_2012 all 90 SNPs were located in a single 3,594 bp recombination event while the other two isolates had SNPs distributed equally across the entire plasmid.

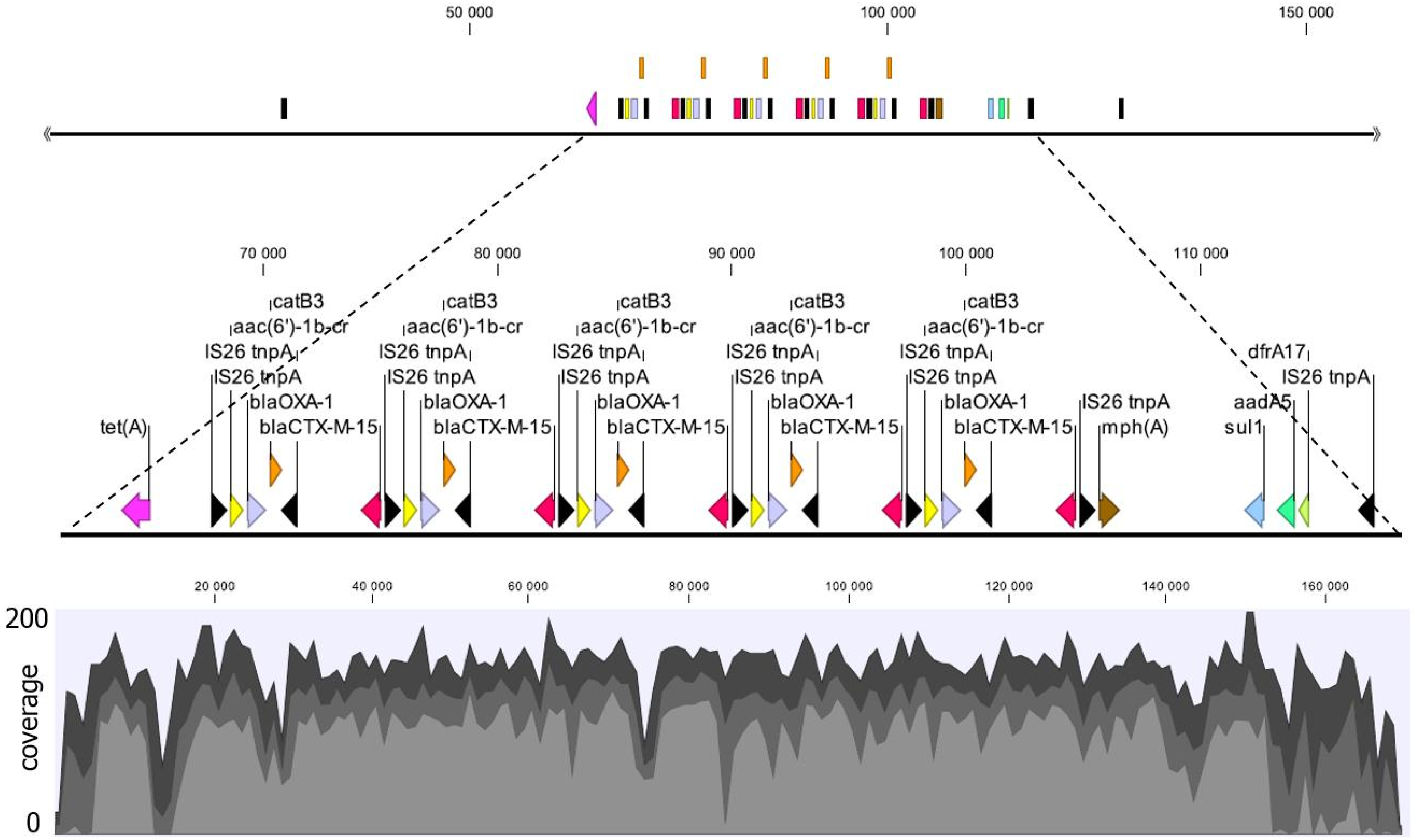

Figure S3. Schematic drawing of the 157,978 bp resistance plasmid from isolate B1527-PB_2012. The resistance genes were located in a 50kb resistance cassette consisting of repetitive gene cassettes including the genes *bla*$_{CTX-M-15}$, aac(6')-1b-cr, *bla*$_{OXA-1}$ and catB3 flanked by IS26 tnpA. Assembly of short-read data against PacBio plasmid with even coverage over repetitive regions is shown in the lower part of the figure.

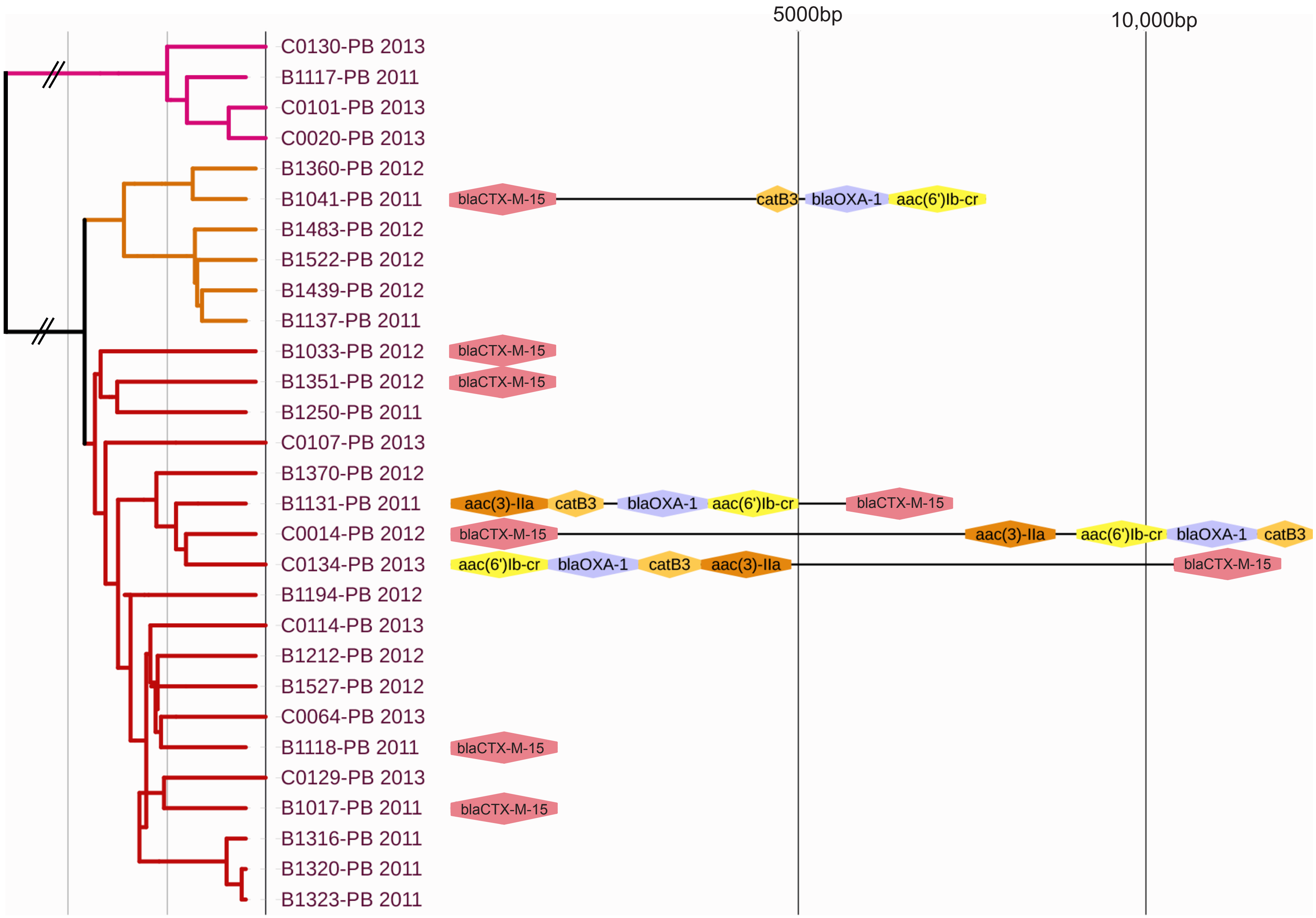

Figure S4. Comparison of chromosomally encoded resistance gene placement in Swedish ST131 isolates. In total eight isolates had chromosomal resistance genes and all of these had the *bla*$_{CTX-M-15}$ gene.
